# A Portfolio Analysis of Culturally Tailored Trials to Address Health and Healthcare Disparities

**DOI:** 10.3390/ijerph15091859

**Published:** 2018-08-28

**Authors:** Marisa Torres-Ruiz, Kaitlynn Robinson-Ector, Dionna Attinson, Jamie Trotter, Ayodola Anise, Steven Clauser

**Affiliations:** Patient-Centered Outcomes Research Institute (PCORI), Washington, DC 20036, USA; kector@pcori.org (K.R.-E.); dionna.attinson@gmail.com (D.A.); jtrotter@pcori.org (J.T.); aanise@pcori.org (A.A.); sclauser@pcori.org (S.C.)

**Keywords:** Patient-Centered Outcomes Research Institute, cultural tailoring, disparities, intersectionality, comparative clinical effectiveness research

## Abstract

In 2010, Patient-Centered Outcomes Research Institute (PCORI) was authorized by Congress to improve the quality and relevance of evidence available to help patients, caregivers, employers, insurers, and policy makers make better-informed health decisions. We conducted a qualitative analysis of behavioral health trials in the PCORI Addressing Disparities portfolio to examine cultural tailoring strategies across the following priority populations: racial and ethnic minorities, rural populations, people with low-income or low socioeconomic status, individuals with disabilities, people with low health literacy, and lesbian, gay, bisexual, and transgender (LGBT) communities. The *Common Strategies for Enhancing Cultural Appropriateness* model was used to examine cultural tailoring strategies within trials. We hypothesized increased intersectionality within a patient population at risk for disparities would correlate with the dosage and type of cultural tailoring strategies applied. Thirty-three behavioral health trials applied cultural tailoring strategies and a majority of trials (n = 30) used three or more strategies. Trends in cultural tailoring were associated with certain racial and ethnic groups; however, increased use of tailoring was not associated with the number of priority populations included in a trial. The PCORI Addressing Disparities portfolio demonstrates how a range of cultural tailoring strategies are used, within comparative clinical effectiveness research trials, to address the needs and intersectionality of patients to reduce health and healthcare disparities.

## 1. Introduction

Despite substantial improvements in several national health indicators, disparities in health and healthcare outcomes remain pervasive in the United States [1]. Although addressing disparities is a priority for local, state, and federal policy, as well as care delivery settings, it is estimated that the economy loses approximately $309 billion per year due to the direct and indirect costs of disparities [1]. In the United States, populations at risk for disparities have a disproportionate risk of poorer health outcomes, experiencing lower quality care, and receiving differential treatment across a range of diseases and health services [2,3]. Disparities may be viewed through the lens of race and ethnicity, income or socioeconomic status, geographic location, functional status, sexual orientation, and other factors [4]. 

Disparities can be further compounded by intersectionality. Intersectionality recognizes that people have overlapping identities and experiences that do not exist independently, but are interconnected to create a unique convergence of identity [5]. For example, LGBT patients who also identify as racial and ethnic minorities have an even higher risk of poor health outcomes [6]. Similarly, minorities with disabilities experience additional disparities, prejudice, discrimination, economic barriers, and difficulties accessing care, resulting in a “double burden” [7]. Clinicians may lack an understanding of how race and ethnicity further influence the experiences of their patients who may also have a disability or low-income [6]. Within the healthcare system, most clinicians do not have significant experience addressing issues at the intersection of identities, which can influence communication and clinical care.

Populations at risk for disparities have reported significant barriers to receiving culturally competent care that reflects the intersection of their respective identities [6]. As patient populations become more diverse, the implementation of culturally appropriate interventions to address disparities experienced at the intersection of patients’ identities is critical to the conduct of patient-centered outcomes research. The literature demonstrates the need for cultural tailoring of interventions for populations at risk for disparities [8,9]. In fact, a recent report published by the National Quality Forum calls for the United States public health and healthcare system to implement tailored interventions for populations based on their social risk factors in order to eliminate disparities [10]. Consequently, efforts to improve outcomes for populations at risk for disparities should be informed by cultural attributes including shared values, traditions, customs, and history, as well as beliefs and practices of the population [11]. Evidence suggests that cultural norms contribute to behaviors associated with risk factors for chronic diseases [12,13]. Culturally appropriate and tailored interventions can enhance receptivity to, and acceptance of, health information and programs, which may ultimately improve health care outcomes and reduce disparities [13,14]. However, further research on the most effective approaches to deliver culturally tailored interventions across populations is still needed [10].

Culturally appropriate and tailored interventions require adaptation of strategies, messages, and materials to specific cultural characteristics of the trial population [15]. While culturally tailored strategies vary, evidence suggests that incorporating the culture of the specific population in health programs can be more effective than a one-size-fits-all approach in reducing disparities [16]. As the adoption of cultural tailoring strategies in research increases, it is critical to examine the influence of these strategies and patient intersectionality on the development of interventions and patient-centered outcomes. A prominent model used to assess cultural tailoring is the *Common Strategies for Enhancing Cultural Appropriateness* (Table 1) [17]. This model identifies five strategies that can be used independently or in combination, in both research and practice, to effectively tailor an intervention or program. Strategies include less intensive methods, such as peripheral strategies, where materials are created to promote relevance among participants, to more intensive methods, such as sociocultural strategies, that integrate cultural norms and behaviors in health messages to increase significance.

Engaging patients to ensure that interventions are adapted to address their specific needs is central to Patient-Centered Outcomes Research Institute’s (PCORI’s) mission. PCORI funds comparative clinical effectiveness research (CER) that addresses important health and healthcare-related dilemmas faced by patients, caregivers, clinicians, and health systems. By comparing two or more interventions for which evidence of efficacy already exists, and by engaging key patients and stakeholders throughout the research process, PCORI-funded trials have the potential to accelerate the dissemination and implementation of research findings in real-world settings [18]. PCORI, through its Addressing Disparities research portfolio, has made significant investments in trials that conduct CER to address health and healthcare disparities. As of June 2018, PCORI has funded over $247 million (79 trials) in its Addressing Disparities research portfolio that aim to reduce or eliminate disparities in health and healthcare outcomes. PCORI identifies thirteen populations at risk for disparities; however, the specific populations emphasized in the Addressing Disparities portfolio include racial and ethnic minorities, rural populations, people with low-income, individuals with disabilities, LGBT communities, and people with low health literacy (including low numeracy and limited English proficiency) [19].

This paper presents data from behavioral health trials, within the PCORI Addressing Disparities portfolio, that use cultural tailoring strategies to address disparities and improve patient-centered outcomes across a range of conditions. The paper identifies cultural tailoring strategies used in the trials and further assesses trials that address intersectionality. Lastly, the paper draws implications from this review for future research.

## 2. Materials and Methods

Clinical trials were selected from the PCORI Addressing Disparities research portfolio to examine the association between cultural tailoring and populations at risk for health and healthcare disparities. Trials in the analysis: (1) used a behavioral health intervention; (2) aimed to improve outcomes in a population at risk for disparities; and (3) deployed cultural tailoring strategies. A qualitative portfolio analysis was performed to examine descriptive elements of the behavioral health portfolio. Trials using behavioral interventions were chosen for this analysis based on evidence suggesting that behavioral interventions are integral when addressing disparities in health [20,21]. Behavioral health interventions were defined as trials that used intervention components to change a study participant’s behavior. These interventions included, for example, psychotherapy or lifestyle counseling. Population(s) included in each trial represent those targeted in the Addressing Disparities research portfolio. Culture was defined as shared thoughts, actions, beliefs, and institutions related to “racial, ethnic, religious, spiritual, linguistic, biological, socioeconomic, geographical or sociological characteristics racial, ethnic, religious, spiritual, linguistic, biological, socioeconomic, geographical or sociological characteristics” [22]. For the purpose of this analysis, cultural tailoring was defined as the adaptation of the study design, materials, and other components of the intervention to reflect cultural needs and preferences at the population level [11]. The trials that met the inclusion criteria were examined against the *Common Strategies for Enhancing Cultural Appropriateness* model to identify the use of cultural tailoring strategies [17]. This tailoring model was selected to conduct the analysis due to its widespread application and notoriety. Publicly available abstracts, as well as internal documents available to PCORI staff, such as study protocols, and biannual progress reports, were reviewed and coded for each intervention according to the cultural tailoring methods in the model. Each trial was assigned and analyzed by a single coder familiar with the trial and the Addressing Disparities portfolio. After completion, all coders met to review the coding collectively and any questions regarding coding were discussed and reconciled to ensure accuracy.

## 3. Results

### 3.1. Health Conditions Represented across Culturally Tailored Trials

Thirty-three trials met the inclusion criteria for this analysis. Trials within the portfolio deployed cultural tailoring strategies across a broad range of health conditions. Approximately 21 percent of interventions were designed to improve health outcomes related to nutritional and metabolic disorders, 18.2 percent addressed mental/behavioral health, and 15.2 percent focused on respiratory diseases (Table 2).

### 3.2. Application of Cultural Tailoring Strategies

**Figure 1:**
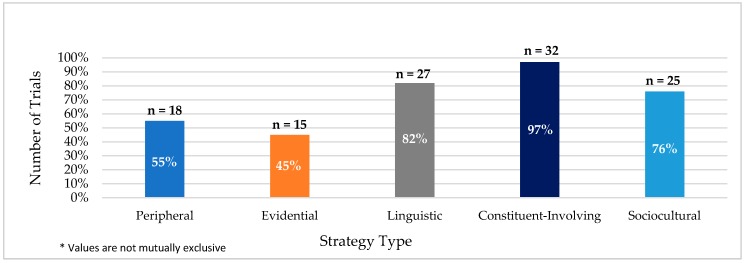
Use of cultural tailoring strategies among trials.

All trials in this analysis used two or more cultural tailoring strategies, while the majority of trials used three or more strategies. Thirty-six percent and 30 percent of trials implemented three or four cultural tailoring strategies, respectively. Ninety-one percent of trials used three or more strategies. The most utilized strategies included constituent-involving, linguistic, and sociocultural. Ninety-seven percent of trials implemented constituent-involving strategies, 82 percent of trials implemented linguistic strategies, and 76 percent of trials implemented sociocultural tailoring strategies (Figure 1). These three strategies were used in combination across 64 percent of trials. The least used cultural tailoring methods included peripheral (55 percent) and evidential (45 percent).

### 3.3. Cultural Tailoring Strategies across Populations at Risk for Disparities

**Figure 2:**
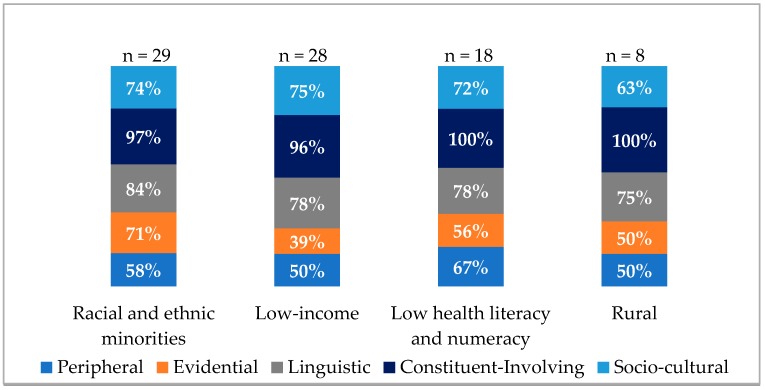
Cultural tailoring strategies by priority population.

When classified by the six Addressing Disparities research priority populations, 88 percent of trials addressed racial and ethnic minorities, 85 percent of trials addressed low-income populations, 55 percent addressed low health literacy and numeracy, and 24 percent addressed rural populations (Figure 2). Within the Addressing Disparities portfolio, no trials targeting LGBT communities or individuals with disabilities applied cultural tailoring strategies. Populations represented within trials were not mutually exclusive. 

Across the four priority populations where cultural tailoring strategies were used, sociocultural, constituent-involving, and linguistic strategies were the three most applied cultural tailoring strategies. Results for the implementation of cultural tailoring among the four priority populations are shown in Figure 2. For rural populations, 50 percent of trials used peripheral and evidential strategies, 75 percent used linguistic strategies, 100 percent used constituent-involving strategies, and 63 percent used sociocultural tailoring strategies. For trials focused on racial and ethnic minorities, 58 percent of interventions used peripheral strategies, 71 percent used evidential strategies, 84 percent used linguistic strategies, 97 percent used constituent-involving strategies, and 74 percent used sociocultural tailoring strategies.

### Cultural Tailoring Strategies among Racial and Ethnic Minorities

Eighty-eight percent of the trials focused on racial and ethnic minorities (n = 29), with 31 percent (n = 9) targeting African Americans, 31 percent (n = 9) targeting Hispanics and Latinos, 14 percent (n = 4) targeting Asians and Pacific Islanders, and 10 percent (n = 3) targeting American Indians and Alaska Natives. Fourteen percent of the trials (n = 4) targeted multiple racial and ethnic populations (e.g., Hispanics and Latinos and African Americans). The remaining trials did not have a specific emphasis on a racial or ethnic population.

**Figure 3:**
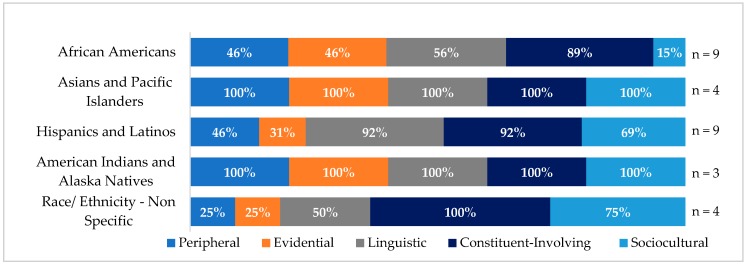
Cultural tailoring strategies by race and ethnicity.

One-hundred percent of trials targeting Asians and Pacific Islanders and American Indians and Alaska Natives used all five cultural tailoring strategies (Figure 3). In trials that targeted Hispanics and Latinos, 46 percent used peripheral strategies, 31 percent used evidential strategies, 92 percent used linguistic and constituent involving strategies, and 69 percent used sociocultural tailoring strategies. In trials that targeted African Americans, 46 percent used peripheral and evidential strategies, 56 percent used linguistic strategies, 89 percent used constituent-involving strategies, and 15 percent used sociocultural tailoring strategies. Among trials that focused on specific racial and ethnic groups, trials including Hispanics and Latinos deployed the fewest evidential tailoring approaches (31 percent), followed by African Americans (46 percent). Compared to other racial and ethnic groups, fewer trials targeting African Americans used sociocultural (15 percent) and linguistic (56 percent) cultural tailoring strategies. 

### 3.4. Cultural Tailoring Use and Intersectionality

**Figure 4:**
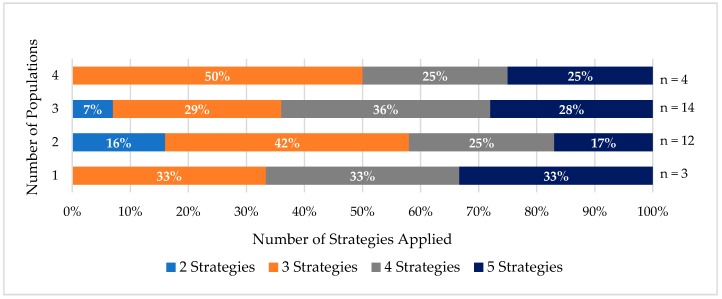
Usage of cultural tailoring among priority populations.

While few trials included only one priority population, over half of the trials enrolled populations with overlapping identities. The number of cultural tailoring strategies used varied when intersectionality was assessed. In trials with two priority populations, 16 percent used two strategies, 42 percent used three strategies, 25 percent used four strategies, and 17 percent used five strategies (Figure 4).

In trials with three priority populations, 7 percent, 29 percent, 36 percent, and 28 percent used two, three, four, and five cultural tailoring strategies, respectively. Ninety-three percent of these trials used three or more strategies.

All trials that enrolled four priority populations deployed three or more cultural tailoring strategies. Fifty percent of these trials used three strategies and 25 percent used four and five strategies. Regardless of the number of populations included in a trial, most trials used three or more strategies to culturally tailor their intervention.

**Figure 5:**
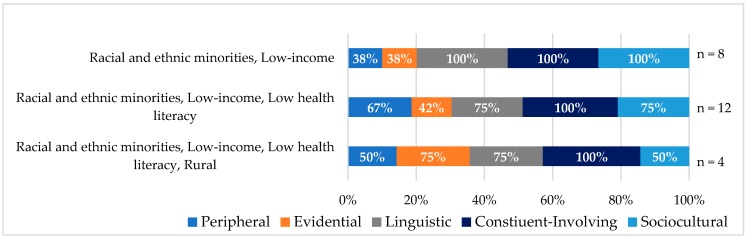
Cultural tailoring use and intersectionality.

Figure 5 highlights the most frequent overlapping identities and the combination of cultural tailoring strategies used among trials. Constituent-involving strategies were used at a similar rate across population groups. Within trials that enrolled racial and ethnic minorities, who also identified as low-income, all used linguistic, constituent-involving and sociocultural cultural tailoring strategies; however, only 38 percent used peripheral and evidential strategies.

In trials with more complex patient populations, such as racial and ethnic minorities with low-income and low health literacy, an increase was observed in the use of peripheral (67%) and evidential strategies (42%). In comparison, racial and ethnic minorities with low-income had fewer of these strategies applied.

Among trials that addressed racial and ethnic minorities who had low-income, low health literacy, and lived in rural areas, evidential and linguistic strategies were used by 75 percent of the trials. Sociocultural strategies were least likely to be implemented in this population, with 50% of trials using this strategy.

## 4. Discussion

Cultural tailoring strategies are frequently applied in behavioral health trials that are part of PCORI’s Addressing Disparities portfolio. Behavioral health trials, which aim to change health related attitudes and behaviors among participants, are likely more amenable to the application of cultural tailoring than drug or device trials. This may be due to the influence culture has on health behaviors [23]. While the use of cultural tailoring strategies in trials varies by racial and ethnic minorities, populations with low-income, populations with low health literacy and numeracy, and rural populations, there is substantial use of multiple strategies across these groups. Further, intersectionality is present among patient populations in more than half of the trials, and highlights the variability in the use of tailoring strategies even among populations that have overlapping identities. While not all trials were designed to address overlapping patient identities, many used cultural tailoring strategies to address the intersections within their enrolled populations. The following sections discuss cultural tailoring use among the largest priority populations in the analysis, considerations of intersectionality, and the relationship between cultural tailoring and patient engagement. Further discussion includes the implications of research into practice, recommendations for future areas of research, and points for consideration and limitations.

### 4.1. Cultural Tailoring among Racial and Ethnic Minorities 

A variety of cultural tailoring strategies were used among trials to meet the needs of racial and ethnic minorities. The heterogeneity of racial and ethnic groups supports the differences in strategies used.

#### 4.1.1. Asians and Pacific Islanders, American Indians, and Alaska Natives 

Compared to other racial and ethnic groups in this analysis, all trials targeting Asians, Pacific Islanders, American Indians, and Alaska Natives applied all five cultural tailoring methods. This outcome may be attributed to several factors. Asian Americans, Pacific Islanders, American Indians, and Alaska Natives are extremely diverse in language, English proficiency, socioeconomic status, health status, cultural identity, and knowledge and beliefs regarding causes of disease and modes of treatment [24]. To address the complexities in these populations and ensure the development of interventions, strategies, and materials that meet their needs, the use of all tailoring strategies were applied in trials. Given that 32 Asian languages are spoken within the United States and over 20 languages are spoken among American Indians and Alaska Natives, trials recognized the need to address linguistic barriers [25,26,27]. Additionally, many Asian cultures historically practice Eastern medicine and American Indians and Alaska Natives might also rely on traditional healing practices [28]. Trials may have implemented sociocultural tailoring strategies to incorporate these cultural medical beliefs and practices into interventions. Constituent-involving cultural tailoring strategies have also been shown to be of importance for Asians, Pacific Islanders, American Indians, and Alaska Natives, as interventions that are not designed in partnership with the community are less likely to be successful for these communities [29].

The PCORI-funded trial, *Extended Family Model of DSME to Reduce Disparities in a US Pacific Islander Community* (see Supplementary Materials), provides an example of how all five cultural tailoring strategies can be applied to the development and implementation of trial components. The trial aims to recruit 221 Marshallese, 18 years of age or older, with type 2 diabetes and their family members. This trial compares eight sessions of the standard diabetes self-management education (DSME) intervention delivered by a healthcare professional to eight Family Model DSME sessions delivered in Marshallese. To ensure that the intervention is culturally appropriate and designed to help Marshallese individuals with type 2 diabetes maintain healthy blood glucose levels, a community-based participatory research (CBPR) approach was used to modify an existing DSME intervention [30]. The trial’s Curriculum Adaptation Team, which consists of Marshallese community members, researchers, and clinical experts, identified important cultural factors to incorporate into the intervention. The trial is designed to include Marshallese beliefs, attitudes, religion, family structures, and eating habits [31]. For instance, community representatives suggested honoring spiritual beliefs by integrating the idea that God provides strength to make healthy choices [31]. Researchers also use a collective family framework to ensure that a patient’s entire family participated in diabetes management activities, such as glucose monitoring.

#### 4.1.2. Hispanics and Latinos

The majority of trials (92%) within the subset of interventions targeting Hispanics and Latinos use linguistic tailoring to address the language and literacy needs of this population. In these trials linguistic tailoring goes beyond the translation of intervention materials by incorporating bilingual staff to bridge language barriers between patients, research staff, and providers to facilitate improved care. Sociocultural tailoring is also a common tailoring strategy among trials targeting Hispanics and Latinos. Sixty-nine percent of trials in this analysis rely on sociocultural tailoring to integrate the cultural values, norms, religious beliefs, and behaviors of this target group [17]. It was anticipated that more trials would implement sociocultural tailoring strategies within this population; however, researchers may consider linguistic tailoring strategies sufficient to address the needs of this population. Trials that implement both linguistic and sociocultural strategies likely understand the important distinction between translation of language and the incorporation of cultural beliefs and values. Research on the effectiveness of cultural tailoring supports the use of these two strategies in combination, as it results in significantly stronger effects, compared to other forms of tailoring [32].

One PCORI trial that implements linguistic and sociocultural tailoring strategies is *Programa Esperanza* (see Supplementary Materials) [33]. This psychosocial intervention is a trial for Spanish-speaking Latino patients, 55 years of age or older, with depression and multiple medical conditions [33]. The control arm of the intervention applies enhanced usual care, which consists of referrals to specialty mental health services and distribution of educational materials on depression and depression treatment to patients. The intervention administers “Problem-Solving Treatment” across eight weekly sessions, in addition to three booster sessions [33]. Patients are matched with a bilingual health professional to accommodate their linguistic and cultural needs. This concordance is maintained throughout counseling sessions and follow-up timepoints to more effectively respond to depressive symptoms. As part of the intervention, patients generate action plans that identify solutions to potential life challenges and participate in activities that alleviate stress. Both the action plans and activities often integrate sociocultural components, such as religion. The use of these cultural considerations effectively adapts the intervention for the intended population.

#### 4.1.3. African Americans

African Americans represent a diverse population with variation in dialect, literacy, socioeconomic status, and geographical location. Overall, trials that target African Americans use the least amount of sociocultural (15%) and linguistic tailoring (56%) strategies. The use of sociocultural strategies within clinical trial research ensures the perspectives of a population are respected and adequately addressed within the study design. The limited use of sociocultural tailoring strategies among African Americans may be due to the belief that constituent-involving strategies are sufficient to address sociocultural needs. Although both sociocultural tailoring and constituent-involving strategies rely on the engagement of individuals from the target population, sociocultural tailoring is a more immersed form of tailoring which integrates the values and beliefs of a population into the intervention. As African American communities are not homogeneous and include complex and varying cultures, use of sociocultural tailoring may be critical for improving interventions delivered to this group. Anecdotal information from discussions with research teams suggest that sociocultural tailoring strategies may have been implemented in several trials a posteriori when faced with challenges related to recruitment or retention; however, interviews with research teams and other collaborators were not included in the documentation for this analysis. 

Linguistic tailoring strategies seek to make interventions and their accompanying materials and messages more accessible by providing them in the dominant or native language of the target group [17]. Linguistic tailoring includes translating materials into a specific language, as well as calibrating content to the health literacy levels of the intended population. Given that low health literacy negatively impacts health indicators such as hospitalization rates, emergency care use, medication adherence, and the ability to interpret labels and health messages, trials targeting African Americans should tailor interventions to address low literacy rates [34,35,36]. The limited use of linguistic tailoring strategies among trials that target African Americas may be attributed to trials primarily addressing other barriers, such as access to care. Since low health literacy is prevalent among African Americans, linguistic tailoring strategies should be considered to address disparities in health and healthcare in this population [34].

### 4.2. Cultural Tailoring among Populations with Low-Income

Trials targeting individuals with low-income represent the second largest priority population in this analysis (n = 28). Low-income or socioeconomic status is a prominent indicator of health and often associated with health risk behaviors [37]. To address the challenges experienced by individuals with low-income, researchers implemented a combination of cultural tailoring strategies, with constituent involvement being the most frequently used strategy (96 percent). Gathering knowledge and shared experiences from a group via constituent involvement can be an effective way to build trust among patient populations and better understand behaviors of a group.

Evidential strategies were the least applied cultural tailoring method (39 percent) in trials targeting populations with low-income. Evidential strategies include sharing epidemiological data with patients, such as their risk for acquiring certain communicable or chronic diseases. The limited use of evidential strategies among populations with low-income may be due to the association between education and income and the perceived challenges of communicating complex health information to patients [38]. Lack of education, income, and one’s occupation can directly impact an individual’s risk for disease. For individuals with low-income, evidential strategies present an opportunity to create effective interventions that provide targeted health information to patients with high risk for experiencing health and health care disparities.

### 4.3. Considerations of Intersectionality across Cultural Tailoring Approaches

A patient-centered approach to cultural tailoring considers the intersectionality that may exist within populations at risk for disparities. As an emphasis has recently been placed on treating multiple chronic conditions holistically, rather than on a disease specific level, the same is necessary when treating a patient with intersections of identities.

Intersectionality is present within the Addressing Disparities portfolio, as a majority of the trials (n = 30) in this analysis focus on populations that identify with two or more identities. Despite high occurrence of intersectionality among populations, few trends emerged in cultural tailoring strategies for specific intersections of identities. One trend observed was the use of sociocultural tailoring in 50 percent of trials targeting racial and ethnic minorities who live in rural settings, demonstrate low health literacy and have low-income. When compared to other groups with intersections of identities, this particular group has the lowest rate of sociocultural tailoring. This may be indicative of challenges researchers face when applying sociocultural strategies to meet the needs of complex populations.

### 4.4. Connection between Cultural Tailoring and Patient Engagement

In 2010, PCORI was authorized by Congress to improve the quality and relevance of evidence available to help patients, caregivers, employers, insurers, and policy makers make better-informed health decisions. Patient engagement, a guiding principal which drives the PCORI research agenda, establishes a partnership among practitioners, patients, and their families to ensure that decisions respect patients’ wants, needs, and preferences, and that patients have the education and support they need to make decisions and participate in their own care [39]. PCORI works to engage patients and stakeholders in every phase of research, with a goal of better understanding treatment effects, which may differ across patient populations [18].

Within the Addressing Disparities portfolio, constituent-involving strategies, where members of the community are included and engaged in intervention activities, are the most utilized across trials. Constituent-involving strategies, which have been identified as one of the more straightforward strategies to increase cultural competency, are represented in 97 percent (n = 32) of the trials. These trials include patients of the target population as advisory board members or intervention staff. The high rate of constituent-involving strategies is likely due to the requirement PCORI places on investigators to include patient and stakeholder engagement throughout the research process. In fact, one systematic review has shown that studies with significant findings commonly report constituent-involving strategies, or patient engagement strategies, within their intervention [40]. Of note, three percent (n = 1) of the trials do not use constituent-involving strategies. This trial engages patients and stakeholders; however, they do not represent patients from the intended target population.

### 4.5. Implications of Research into Practice

In recent years, incorporating cultural beliefs and values into practice has been integrated into national policy. For example, the Federal Office of Minority Health’s National Standards for Culturally and Linguistically Appropriate Services in Health Care (CLAS) aims to improve health care quality and advance equity by providing standards for health organizations to serve diverse communities throughout the United States [41]. Its principal standard charges healthcare organizations to “provide effective, equitable, understandable and respectful quality care and services that are responsive to diverse cultural health beliefs and practices, preferred languages, health literacy and other communication needs” [41].

With increasing focus on health policies that advance equity, there is recognition of the influence that intersectionality has on health outcomes [40]; however, few methods have been created to translate the theory of intersectionality into practical strategies to be implemented by decision makers, researchers, and policymakers [42]. Further, to date, there are limited policies that take intersectionality into consideration. Findings from PCORI-funded research on cultural tailoring strategies may contribute to the body of evidence that informs policy and practice on how different strategies of cultural tailoring influence health outcomes across conditions, settings, populations with existing intersectionality, and other variables.

### 4.6. Recommendations for Future Areas of Research

Two priority populations, individuals with disabilities and LGBT communities, are not represented in this analysis of behavioral health trials in PCORI’s Addressing Disparities portfolio. Within these populations, more research is needed to understand the most effective type of cultural tailoring strategies to ensure that interventions meet the needs of these patients. Research should assess how intersectionality influences health behavior and what forms of cultural tailoring are best suited to address the “double, triple, or quadruple burdens” experienced in these populations [43,44]. With the influence that sociocultural tailoring strategies have on study effectiveness and improvements in health outcomes, future trials should consider prioritizing the implementation of this strategy. Future research should address the impact that sociocultural strategies have on research and outcomes, as well as the influence that the combination of constituent-involving and sociocultural strategies can have in culturally tailored trials.

### 4.7. Points for Consideration and Limitations

Within the field of cultural tailoring there exists two approaches to constructing culturally appropriate health messaging and materials: cultural tailoring and cultural targeting. Though these terms are often used interchangeably, the main distinction between the two approaches is the scale at which they are implemented. Cultural tailoring strategies are often applied on an individual level while cultural targeting techniques are commonly carried out at a population level [11]. The majority of the trials in this analysis engage in cultural targeting, however, there are a small number of studies that engage in cultural tailoring. We applied the term tailoring for this analysis because of the widespread familiarity of the phrase in the field of health services research. Furthermore, while this analysis uses the *Common Strategies for Enhancing Cultural Appropriateness* model, other models for cultural tailoring exist [17,45,46]. If this portfolio of trials were analyzed against an alternate cultural tailoring model, the results of this analysis could vary. As the current analysis was limited to thirty-three trials, a larger sample of trials could provide additional information and context for the most effective cultural tailoring strategies for specific populations.

While the PCORI Addressing Disparities portfolio fills a known gap in the application of cultural tailoring methods beyond racial and ethnic groups, this analysis is limited in how intersectionality is assessed. This analysis was confined to analyzing intersectionality within the six Addressing Disparity priority populations; however, there are many other social and cultural identities that also impact health and healthcare outcomes. Additionally, racial and ethnic minorities are often defined too broadly. Racial and ethnic minorities as a broad category and even specific racial or ethnic groups may have existing heterogeneity in language, culture, health, and life circumstances [11]. There are instances where cultural traits are not equally valued within groups that appear to be homogeneous and where common beliefs and values are shared between two distinct groups [11]. This can be a challenge when identifying and addressing the intersections within a population in research trials and in healthcare practice. Lastly, most of the trials are ongoing and are not expected to have final research results available for the next three years. Once these trials are complete and their results are published, this analysis can be expanded to address the influence of cultural tailoring on patient-centered, patient-reported, and clinical health outcomes.

## 5. Conclusions

The PCORI Addressing Disparities portfolio demonstrates how a range of cultural tailoring strategies are used within CER trials to address the needs and intersectionality of patients to reduce health and healthcare disparities. While several cultural tailoring strategies are used in trials, more work is needed to ensure that all research focusing on populations at risk for disparities incorporates as many strategies as appropriate in their trials. Adopting an approach that uses the five strategies in the *Common Strategies for Enhancing Cultural Appropriateness* model aligns with principles of patient-centeredness and patient engagement and can result in improved patient response to care, compliance, and connectivity with the health system.

## Figures and Tables

**Table 1:** Common strategies for enhancing cultural appropriateness [17].

Strategy	Definition
Peripheral	Design of materials to appeal to a given group
Evidential	Enhance perceived relevance of health issue by presenting epidemiological evidence to participants
Linguistic	Provide materials and services in dominant and/or native language of the target group
Constituent-Involving	Draw on the experience of the group, including members of the indigenous population (i.e., community health workers)
Sociocultural	Use of cultural values, beliefs and behaviors to provide context and meaning to health messages

**Table 2:** Health conditions represented in the analysis.

Condition	Number of Trials, n (%)
Nutritional and Metabolic Disorders	7 (21.2%)
Mental/Behavioral Health	6 (18.2%)
Respiratory Diseases	5 (15.2%)
Cardiovascular Health	4 (12.1%)
Cancer	3 (9.1%)
Infectious Diseases	2 (6.1%)
Reproductive and Perinatal Health	2 (6.1%)
Kidney Disease	1 (3%)
Multiple/Co-Morbid Chronic Conditions	1 (3%)
Muscular and Skeletal Disorders	1 (3%)
Neurological Disorders	1 (3%)
Total	n = 33

## Supplementary Materials

The following are available online at http://www.mdpi.com/1660-4601/15/9/1859/s1: an accompanying video and project summary of the *Programa Esperanza* trial and project summary of the *Extended Family Model of DSME to Reduce Disparities in a US Pacific Islander Community* trial.
