# Supplementary material for: A Portfolio Analysis of Culturally Tailored Trials to Address Health and Healthcare Disparities
Source: Int J Environ Res Public Health. 2018 Aug 28;15(9):1859. doi: 10.3390/ijerph15091859 (PMC6165382; doi:10.3390/ijerph15091859)
Supplement: Supplementary file 1 [file ijerph-15-01859-s001.zip › ijerph-335740-SI/ijerph-355740-supplementary/Aranda Project Summary .pdf]

Treating Depression in Older Latino Adults

Project Summary

This research project is in progress. PCORI will post the research findings on this page within 90 days after the results are final.

What is the research about?

Becoming depressed late in life is a common health problem with serious consequences for many adults, caregivers, and society as a whole. Older adults may have symptoms that differ from younger people's, making it harder to diagnose their depression. Compared with younger people, older adults are less likely to get the care they need for depression and less likely to get better. Also, older adults are more likely to commit suicide. Doctors sometimes don't know the best way to help older people with depression.

Older Latinos have high rates of depression, which affects their everyday lives. Compared with other older adults, they tend to stay depressed longer, and they are less likely to get the best care for depression. This study compares two ways of treating depression in older Spanish-speaking Latino patients:

- Usual care, which includes regular healthcare services plus handouts about depression and depression treatment for older adults
- A program that teaches people about their mental health problems, gives them problem-solving skills, and suggests things they can do to feel better

Who can this research help?

The results of this study may help people, including older Latino adults, their families, and their healthcare providers, make decisions about treatment for depression.

What is the research team doing?

The research team is working with a local health system that offers special care for older patients who have trouble with daily tasks and need care for more than one health problem. The team is recruiting 250 patients to be part of the study.

Half of the patients get usual care for depression, which includes a regular doctor's visit plus handouts about depression and depression treatment for older adults. The other half get one-on-one meetings with trained senior center staff over the course of eight weeks. The meetings use a workbook to teach them about their mental health problems, talk about ways to solve the problems patients face, and help them get back to doing things that make them feel better.

Patients in both groups fill out a survey when the project starts and again after three months, six months, and one year. The surveys ask about what depression symptoms patients have, how they solve problems they have with other people, what habits they have that work well for them, and how being depressed affects their daily lives. The research team is talking to some of these patients to find out what they liked or disliked about the treatment they got.

The team is also working with advisory groups of patients who give input about the study materials and ways to encourage more people to join the study.

Research methods at a glance

Design Element	Description
Design	Randomized controlled trial
Population	English- or Spanish-speaking Latinos age 55 years or older with depression
Interventions/ Comparators	• Problem-solving treatment<br>• Enhanced usual care
Outcomes	Primary: depression<br><br>Secondary: social problem-solving skills, behavioral activation, disability level, physical functioning
Timeframe	12-month follow-up for primary outcome

Project Details

Principal Investigator

Maria P. Aranda, PhD, MPA, MSW

Project Title

Programa Esperanza (Project Hope)

Project End Date

December 2018

Project Status

In progress; Enrollment complete

Board Approval Date

September 2014

Organization

University of Southern California

Year Awarded

2014

Project Type

Research Project

Conditions

Functional Limitations and Disabilities
Mental/Behavioral Health
Multiple/Comorbid Chronic Conditions
Cognitive Difficulty
Depression
Mild Cognitive Impairment

State

California

Funding Announcement

Addressing Disparities

Project Budget

$1,479,449

Study Registration Information

HSRP20152063

NCT02459860

Treating Depression in Older Latino Adults. Patient-Centered Outcomes Research Institute. 2018. Available online: https://www.pcori.org/research-results/2014/treating-depression-older-latino-adults (accessed on 7 June 2018).
