# Supplementary material for: A Portfolio Analysis of Culturally Tailored Trials to Address Health and Healthcare Disparities
Source: Int J Environ Res Public Health. 2018 Aug 28;15(9):1859. doi: 10.3390/ijerph15091859 (PMC6165382; doi:10.3390/ijerph15091859)
Supplement: Supplementary file 1 [file ijerph-15-01859-s001.zip › ijerph-335740-SI/ijerph-355740-supplementary/Aranda Project Video.pdf]

Programa Esperanza (Project Hope)

Principal Investigator: Maria P. Aranda, PhD, MPA, MSW

The primary aim of the study is to test the comparative effectiveness of Programa Esperanza (Project Hope) and enhanced usual care (EUC). Programa Esperanza is a short-term, culturally modified psychosocial intervention for Spanish-speaking Latino patients 55 years of age or older with depression and multiple medical conditions. Our long-term goal is to widely disseminate the results and actionable steps needed to increase the adoption and sustainability of evidence-based behavioral health practices for low-income, Limited-English-speaking geriatric populations.

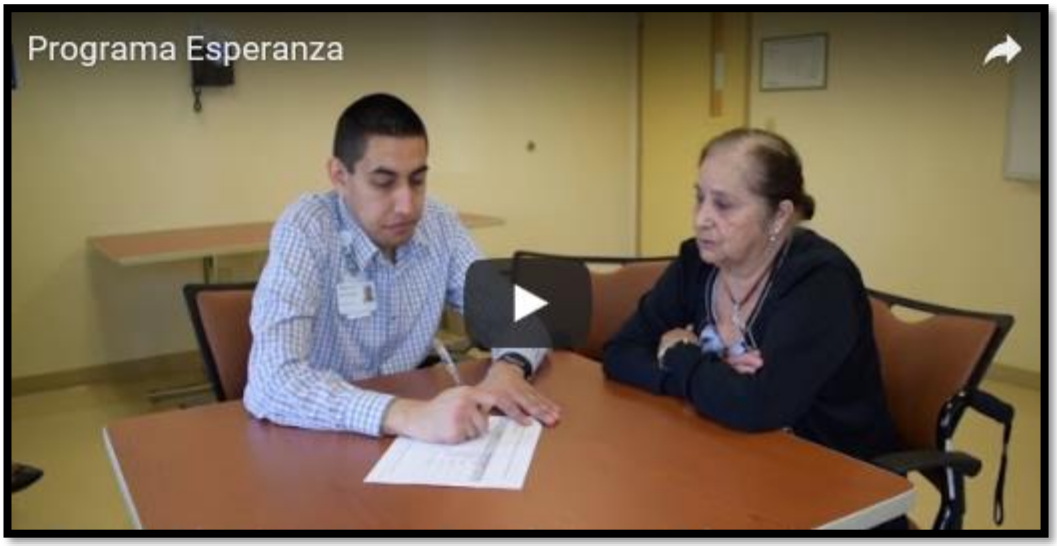

Clinical Intervention Development. 2018. Available online: https://roybal.usc.edu/research-overview/intervention-development/ (accessed on 2 April 2018).
