# Supplementary material for: A Portfolio Analysis of Culturally Tailored Trials to Address Health and Healthcare Disparities
Source: Int J Environ Res Public Health. 2018 Aug 28;15(9):1859. doi: 10.3390/ijerph15091859 (PMC6165382; doi:10.3390/ijerph15091859)
Supplement: Supplementary file 1 [file ijerph-15-01859-s001.zip › ijerph-335740-SI/ijerph-355740-supplementary/Kohler Project Summary.pdf]

Extended Family Model of DSME to Reduce Disparities in a US Pacific Islander Community

Project Summary

The Marshallese population suffers from a significant and disproportionate burden of type 2 diabetes. The rate of type 2 diabetes among the Marshallese is one of the highest of any population group in the world—at least 400 percent higher than the general US population. Prior attempts to implement Diabetes Self-Management Education (DSME) in Marshallese populations have failed to produce even short-term results. On the basis of qualitative interviews using a Community-Based Participatory Research (CBPR) process, the study team hypothesizes that an adapted Extended Family Model of DSME, based on the collectivist, family-centered values of the Marshallese community, will result in better diabetes management outcomes compared with traditional DSME. Using a CBPR approach and an interdisciplinary research team, we will employ the Cultural Adaptation Process to modify and test the DSME as implemented within an Extended Family Model.

Specific aims include (1) adapt the DSME for implementation within an Extended Family Model and (2) Conduct a comparative effectiveness evaluation using a randomized controlled trial design that compares the results of the Extended Family Model of DSME with traditional DSME.

Two local Marshallese organizations, two community co-investigators from the Marshallese community, three community clinics, 31 Marshallese stakeholders, and an extended group of Marshallese, Pacific Islander, and diabetes experts were involved in this application and will continue to be involved throughout the research process.

Project Details

**Principal Investigator**	**Project Status**
Peter O. Kohler, MD	In PCORI Peer-Review Process
**Board Approval Date**	**Project End Date**
July 2014	December 2018
**Organization**	**Year Awarded**
University of Arkansas for Medical Sciences	2014
**State**	**Project Type**
Arkansas	Research Project
**Funding Announcement**	**Conditions**
Addressing Disparities	Nutritional and Metabolic Disorders<br>Diabetes
**Project Budget**	**Study Registration Information**
$2,163,228	HSRP20152031<br>NCT02407132

Extended Family Model of DSME to Reduce Disparities in a US Pacific Islander Community; Patient-Centered Outcomes Research Institute. 2018. Available online: https://www.pcori.org/research-results/2014/extended-family-model-dsme-reduce-disparities-us-pacific-islander-community (accessed on 7 June 2018).
